# Stationary Explosive Trace Detection System Using Differential Ion Mobility Spectrometry (DMS)

**DOI:** 10.3390/s23208586

**Published:** 2023-10-19

**Authors:** Monika Szyposzyńska, Aleksandra Spławska, Michał Ceremuga, Piotr Kot, Mirosław Maziejuk

**Affiliations:** 1 Military Institute of Chemistry and Radiometry, al. gen. A. Chruściela “Montera” 105, 00-910 Warsaw, Poland; a.splawska@wichir.waw.pl (A.S.); p.kot@wichir.waw.pl (P.K.); m.maziejuk@wichir.waw.pl (M.M.); 2 Military Institute of Armoured and Automotive Technology, Okuniewska 1, 05-070 Sulejówek, Poland; michal.ceremuga@witpis.eu

**Keywords:** explosives detection, trace analysis, differential ion mobility spectrometry, personnel portal

## Abstract

Detecting trace amounts of explosives is important for maintaining national security due to the growing threat of terror attacks. Particularly challenging is the increasing use of homemade explosives. Therefore, there is a constant need to improve existing technologies for detecting trace amounts of explosives. This paper describes the design of a stationary device (a gate) for detecting trace amounts of explosives and explosive taggants and the design of differential ion mobility spectrometers with a focus on the gas system. Nitromethane (NM), trimeric acetone peroxide (TATP), hexamine peroxide (HMTD), and explosive taggants 2,3-dimethyl-2,3-dinitrobutane (DMDNB) and 4-nitrotoluene (4NT) were used in this study. Gate measurements were carried out by taking air from the hands, pocket area, and shoes of the tested person. Two differential ion mobility spectrometers operating in two different modes were used as explosive detectors: a mode with a semi-permeable membrane to detect explosives with high vapor pressures (such as TATP) and a mode without a semi-permeable membrane (using direct introduction of the sample into the measuring chamber) to detect explosives with low vapor pressures (such as HMTD). The device was able to detect trace amounts of selected explosives/explosive taggants in 5 s.

## 1. Introduction

Detecting trace amounts of explosives is extremely important due to the growing threat of terrorist attacks. In trace detection, explosive material is detected by identifying microscopic residues of explosive compounds [1,2,3,4,5].

Improvised home-constructed explosives, such as peroxides, which include trimeric acetone peroxide (TATP) and hexamine peroxide (HMTD), are particularly challenging to detect [6,7,8,9,10]. These compounds are readily used by terrorists because they are easy to prepare using commonly available products. In recent years, numerous terrorist attacks have used TATP, including attacks in Paris (2015), Brussels (2016), London (2017), and Sri Lanka (2019).

One of the most widely used techniques for detecting trace amounts of explosives is ion mobility spectrometry (IMS) [11,12,13,14,15,16,17]. The technique has been most extensively developed in commercial trace explosives detection applications in portable detectors, e.g., SABRE 5000 Smith Detection [18], or stationary spectrometers, e.g., IONSCAN Smith Detection [19], operating from swabs of the person.

Entry Scan 4 and Sentinel II are portal explosive trace detection devices [20,21,22] that use IMS technology and can detect a wide range of explosives. Their full measurement cycles, determined from the time a person steps inside the portal to notify the operator, are ~15–20 s. The Sentinel II can detect explosives in 10 s, and the Entry Scan 4’s response time is 13 s. Operating stationary explosive trace detection devices involve taking a sample of the air surrounding the person under investigation and then analyzing the collected sample for residual explosives. An explosive detection device’s gate informs the operator that MW has been detected; however, the MW detection location and the amount of explosive are not identified. These stationary devices, however, have the disadvantage of a rather elaborate gas system and the need to lock up the person under examination in order to detect traces of explosives. The complex gas system requires frequent servicing and therefore significantly increases operating costs. Locking the subject during the test results in the need for staff to direct traffic through the gate, which increases inspection time. It is therefore practically impossible to place this type of equipment in the passenger testing concourses of an airport.

In this paper, we describe a stationary device (a gate) for detecting trace amounts of explosives/explosive taggants. The gate’s detectors used differential ion mobility spectrometry. Two DMS detectors were used: one with a semi-permeable membrane (DMS1) and one without a semi-permeable membrane (DMS2), giving the presented device the ability to detect both low and high vapor pressure explosives. The following compounds were tested: nitromethane (NM), trimeric acetone peroxide, hexamine peroxide, and explosive taggants 2,3-dimethyl-2,3-dinitrobutane (DMDNB) and 4-nitrotoluene (4NT).

The most important features of the presented gateway that distinguish it from other stationary systems are: no closing of the passenger during the measurement; an immediate result on the screen and light lines at the back of the gate; a measuring time of only 5 s; the ability to test all passengers and not just those selected by the system; and low operating costs.

If an explosive/explosive marker is detected, the person being examined is subjected to an additional check with equipment using swabbing methods, e.g., IONSCAN 600.

To our knowledge, this is the first report in the literature showing a stationary explosive trace detection system using differential ion mobility spectrometry. In the literature, gates based on classical ion mobility spectrometry rather than DMS have been described. Furthermore, most gates are closed from the top, whereas in our project, the gate is open and has the ability to screen mainly homemade materials. In addition, thanks to the use of two detectors (with and without a semi-permeable membrane), it is possible to detect both low- and high-vapor-pressure explosives.

## 2. Materials and Methods

### 2.1. DMS Spectrometer’s Principle

**Figure 1:**
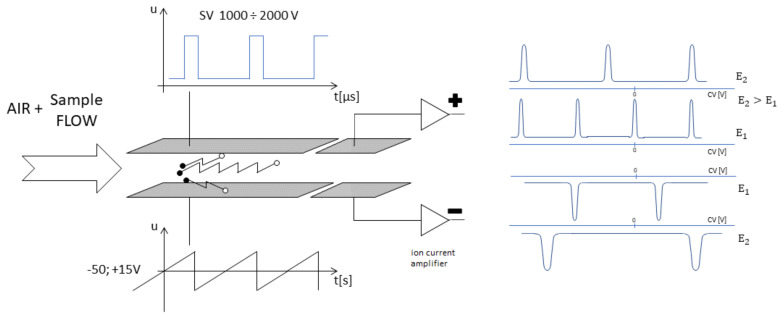
The DMS spectrometer’s principle of operation.

The DMS spectrometer’s principle of operation is shown in Figure 1. The DMS spectrometer is constructed of two parallel plates on which metal electrodes are placed [23,24,25,26]. The electric field is perpendicular to the direction of gas flow. In the spectrometer, there is an area for the ionization of the sample and an area where the ions are separated. Under the influence of the electric field generated in the detector volume, ions are segregated on the collection electrode. The observed segregation of ions present in the flowing gas is due to their different mobility in fields of lower and higher intensity.

The DMS spectrometer can operate with different SV voltages, which allows the use of electric field strengths inside the spectrometer. Different electric field strengths result in different spectrograms, and the position of the peaks changes for each field strength. In some special cases, we observe fragmentation of the ion, and a new peak appears as a result of the fragmentation.

**Figure 2:**
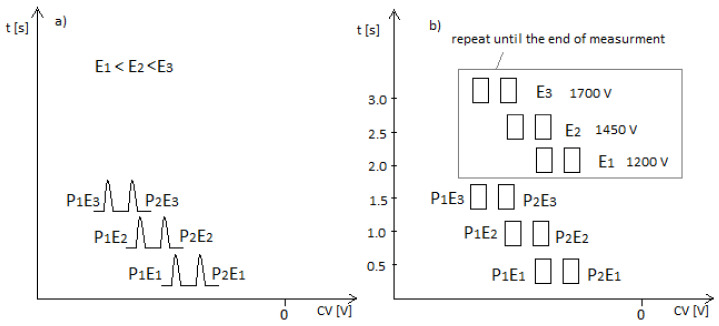
Graphical representation of the registration of spectra in the DMS detector during explosive detection—named multi mode, (**a**) spectra, (**b**) places of increase of ion current.

In the case of the DMS spectrometer used for explosives detection, three SVs are applied to a stationary device, and each voltage is recorded for 0.5 s. The method of recording the spectra is shown in Figure 2.

Figure 2a indicates the peak position (compensation voltage, CV). Subsequent scans are made for successive SV voltages. In our case of the stationary device, this will be 1200, 1450, and 1700 V. When the peaks are mapped on the adjacent axis, we can see that they appear at different locations. In the case of explosive detection, the peaks appear at new locations.

**Figure 3:**
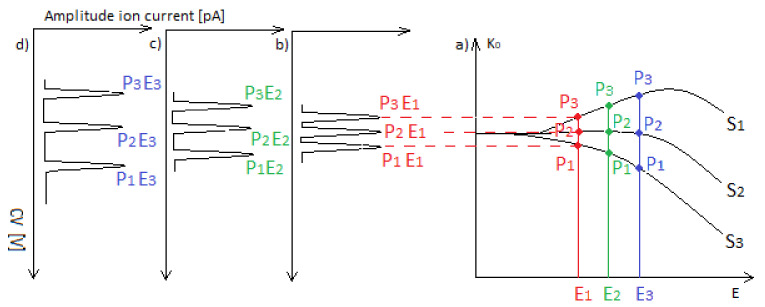
Electric field dependence of mobility for the three substances (**a**) S1, S2, and S3 and three different electric fields, E1, E2, and E3; (**b**–**d**) the spectra’s for high voltage SV separation when the field strengths are E1, E2, and E3, respectively.

Figure 3a shows the changes in ion mobility for three different substances, S1, S2, and S3, and three different electric fields, E1, E2, and E3. Figure 3b–d shows the spectra’s for the DMS spectrometer when, for high voltage SV separation, the field strengths are E1, E2, and E3, respectively. It can be seen that the spectra differ mainly in the position of the peaks. For the highest field, the peak spacing is the largest.

### 2.2. The Gate

**Figure 4:**
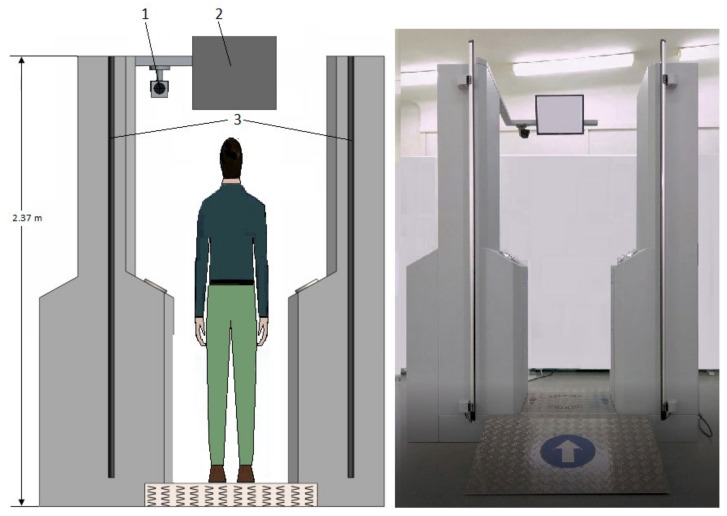
Stationary system scheme: 1—camera; 2—monitor displaying passenger information; 3—curtain to measure the height of a person.

The gate (Figure 4) was equipped with a system for measuring the height of the person being tested. For minors and people shorter than 140 cm, a three-way solenoid valve changed the inlet from W2 to W3 (for the left side) and from W6 to W7 (for the right side).

All inlets fed the gas into a common gas manifold (concentrator), from which the gas was then fed to two DMS spectrometers. All gas lines were heated to 50 °C, and in the line directly before the DMS (gas manifold—concentrator), the temperature reached 90 °C.

Sampling for analysis was conducted from three points on each side placed on the gate:

- The hands (W1, W5);
- Around the pockets (W2, W6);
- The shoes (W4, W8).

**Figure 5:**
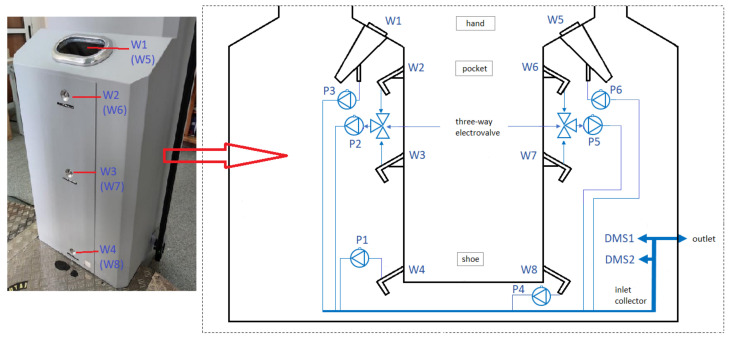
Air intake analysis scheme: 1—hand sample collection system (W1, W5); 2—pocket sample collection system (W2, W6); and 3—shoe sample collection system (W4, W8); P1, P2, P3, P4, P5, P6—pumps.

Sampling for analysis is shown in Figure 5, which presents the sampling air intake analysis scheme.

**Figure 6:**
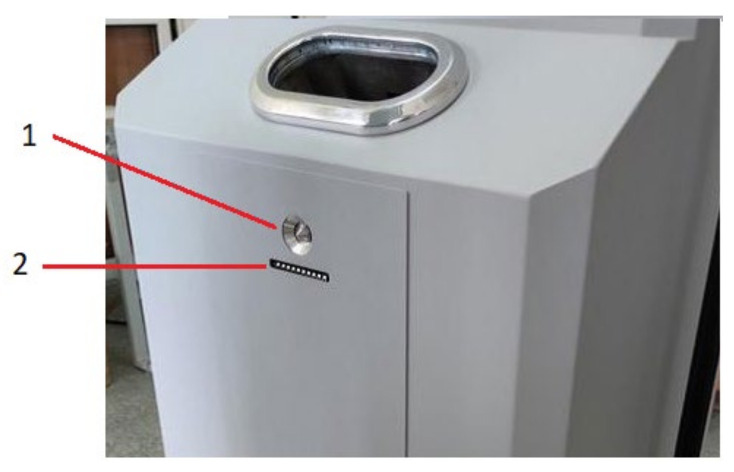
Location of blow-off nozzles relative to the air intake system (1—air intake; 2—blow-off nozzle).

Before each measurement, a blow-off system was activated to blow explosive particles off the clothing or hands of the person being tested. The blow-off system was automatically activated when the measurement procedure began (Figure 6). This system was equipped with a membrane air-drying system and pollen and carbon filters to ensure that the tested air was free of contaminants that could falsify the result. Air is blown out through a blow-off nozzle outlet positioned directly below the sampling nozzles at the level of the shoes, around the pockets, and around the hands (Figure 6). Blow-off nozzles—working pressure: 4 bar.

### 2.3. Sample Preparation

Samples of explosives/explosive taggants applied to 10 cm × 10 cm pieces of cotton material (without dyes or plastic additives) were provided by the Military Institute of Armament Technology (Zielonka, Poland). The surface concentration was approximately 10 µg/cm^2^, and the total surface area with the applied explosive/explosive taggants was approximately 2 cm^2^. Tests using one piece of material with explosive/explosive taggants were repeated several times. The material with the explosive/explosive taggants was planted under the sampling points.

### 2.4. Gas System of DMS1 and DMS2 Spectrometer

#### 2.4.1. Semi-Permeable Membrane Gas System DMS1

**Figure 7:**
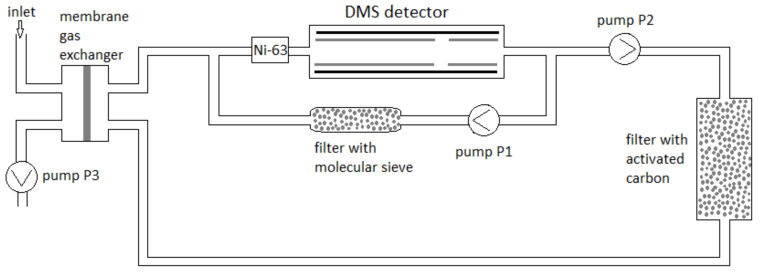
Schematic of the DMS1 detector model’s gas system [27].

The gas system of a detector equipped with a membrane gas exchanger is shown in Figure 7. The system is constructed with the use of three rotation pumps (Thomas) (P1, P2, and P3) with flow rates of 3, 0.5, and 2.5 L/min, respectively. 

The DMS1 detector’s gas system operated as follows: pump P3 drew in air for analysis, which then flowed through a membrane gas exchanger. The DMS chamber system had two air circuits: the DMS chamber’s internal circuit, produced by air pump P1, and external circulation, forced by pump P2. In the DMS chamber’s internal circuit, air passes through molecular sieves to dry residual contaminants. This system stabilized the air flow at 3 L/min through the DMS chamber. The flow rate through the chamber had a decisive effect on the signal value and on the analyzed gas’ residence time in the DMS chamber. To facilitate external circulation, pump P2 fed air to a carbon purification filter, which then passed through a membrane gas exchanger. The ratio of analyzed air to carrier gas was 1:5. The capacity of pump P3 was adjustable from 1% to 100%; its maximum capacity was 2.5 L/min.

#### 2.4.2. Gas System without a Semi-Permeable Membrane (DMS2)

**Figure 8:**
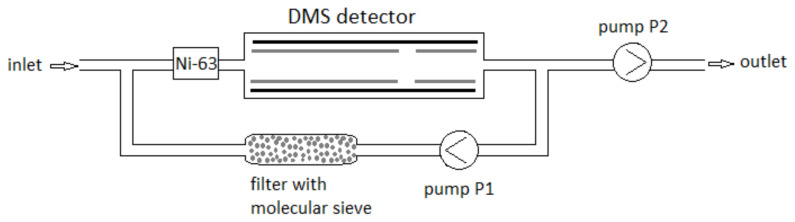
Schematic of the DMS2 detector model’s gas system [27].

A detector without a semi-permeable membrane was used to detect explosives with low vapor pressure. Figure 8 illustrates the gas system of such a detector.

The DMS2 detector’s gas system (without a semi-permeable membrane) was devoid of a P3 pump. Thus, the air circulation was as follows: from the inlet, the air passed into the DMS chamber together with the carrier gas produced during air recirculation through the molecular sieve filter (pump P1); next, pump P2 caused air to flow into the detection system and be blown out after analysis.

Measurements were made using air as a carrier gas and dried with 13 X molecular sieves. The applied molecular sieves clean the gas in the internal circuit of the DMS chamber. The activated carbon raises the gas residual contaminants in the external circulation.

Parameters of both DMS spectrometers:

- Detector temperature: 45 °C;
- Compensating field strength: from −60 V to +15 V;
- Gas flow rate through the detector: 4.0 L/min;
- Carrier gas: purified dry air;
- Separating field strength range: from 400 V to 1700 V;
- Quassi retengular wave, duty factor 27%, frequency 2 MHz;
- Ionization source: radioactive 1.9 GBq ^63^Ni.

DMS chamber:

- Chamber passage channel dimensions: 5 mm × 0.635 mm;
- Length of the control electrode (HV): at least 25 mm;
- Chamber heated with heating resistors;
- The membrane was made of polydimethylsiloxane (PDMS) in the form of a circle 30 mm in diameter and 20 µm thick. PDMS was applied directly to a metal foil with 5 mm diameter holes;
- The chamber is made of ceramic substrates based on thick-layer technology. The distance between the DMS electrode plates is 0.625 mm, and the length of the working electrode is 25 mm. The length of the DMS chamber is 50 mm (with air inlet and outlet).

## 3. Results

**Figure 9:**
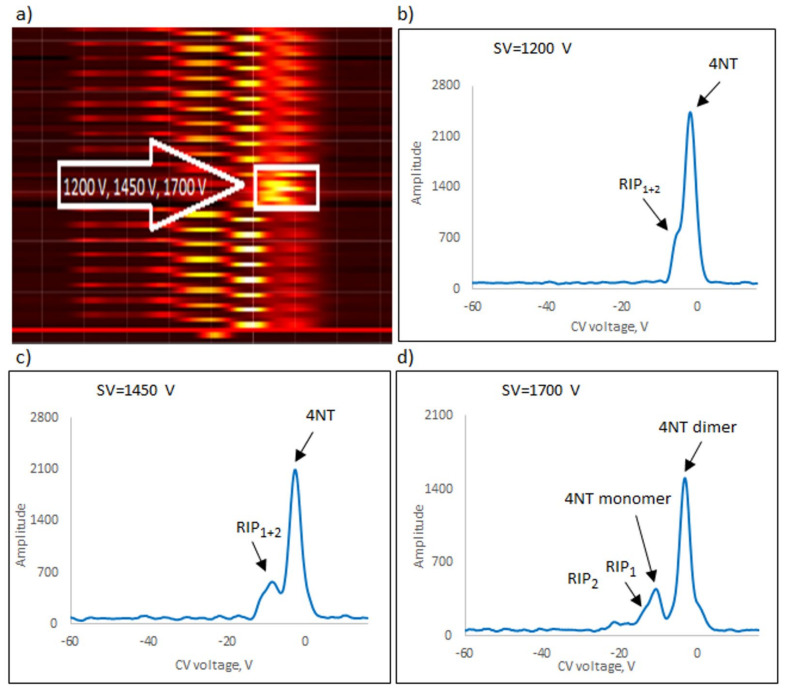
Dispersion plots for positive 4NT ions in (**a**) “multi” mode for voltages 1200 V, 1450 V, and 1700 V and in drift spectra for voltages (**b**) 1200 V, (**c**) 1450 V, and (**d**) 1700 V.

**Figure 10:**
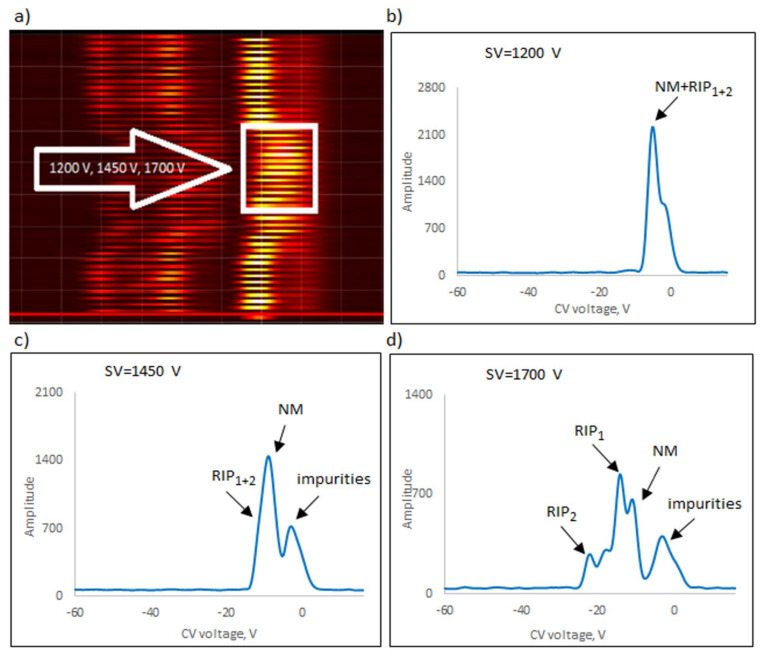
Dispersion plots for positive NM ions in (**a**) “multi” mode for voltages 1200 V, 1450 V, and 1700 V and in drift spectra for voltages (**b**) 1200 V, (**c**) 1450 V, and (**d**) 1700 V.

**Figure 11:**
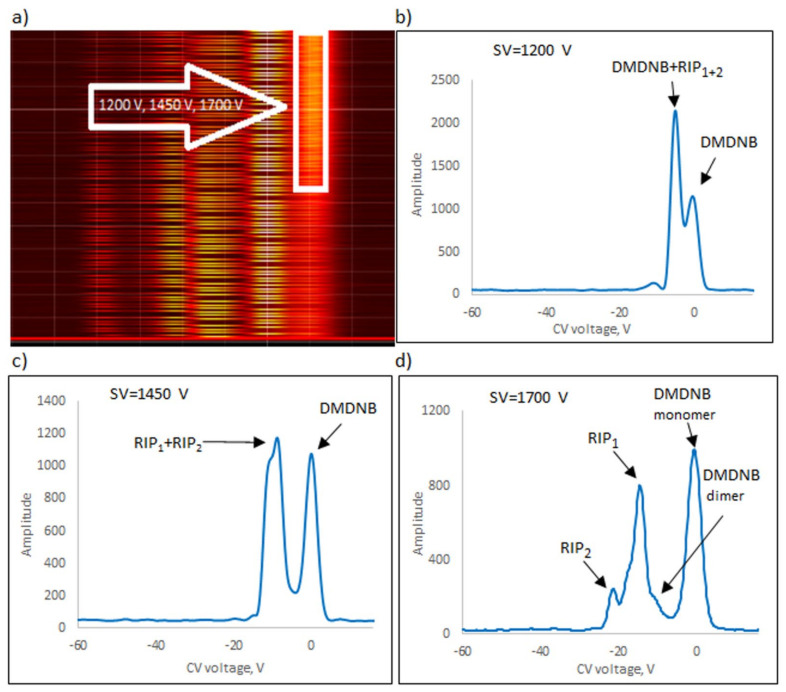
Dispersion plots for positive DMDNB ions in (**a**) “multi” mode for voltages 1200 V, 1450 V, and 1700 V, and in drift spectra for voltages (**b**) 1200 V, (**c**) 1450 V, and (**d**) 1700 V.

**Figure 12:**
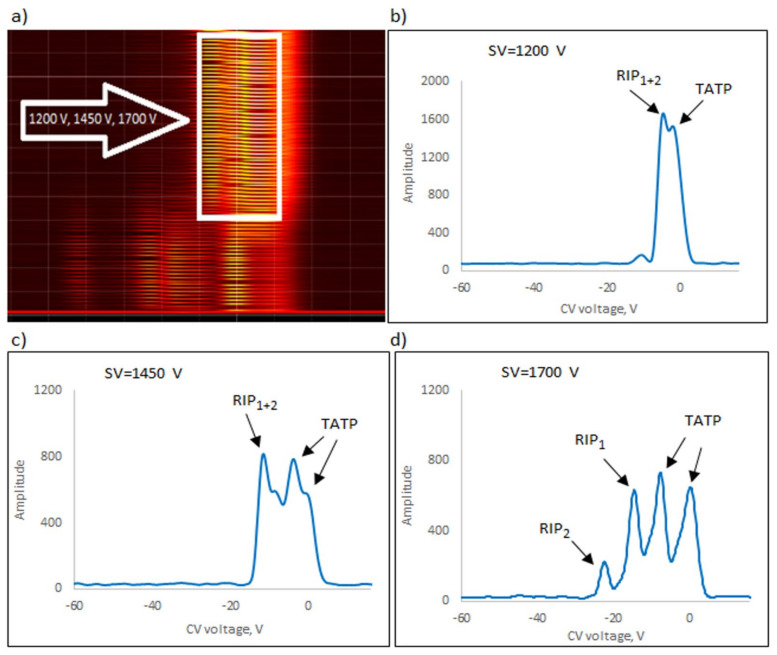
Dispersion plots for positive TATP ions in (**a**) “multi” mode for voltages 1200 V, 1450 V, and 1700 V and in drift spectra for voltages (**b**) 1200 V, (**c**) 1450 V, and (**d**) 1700 V.

**Figure 13:**
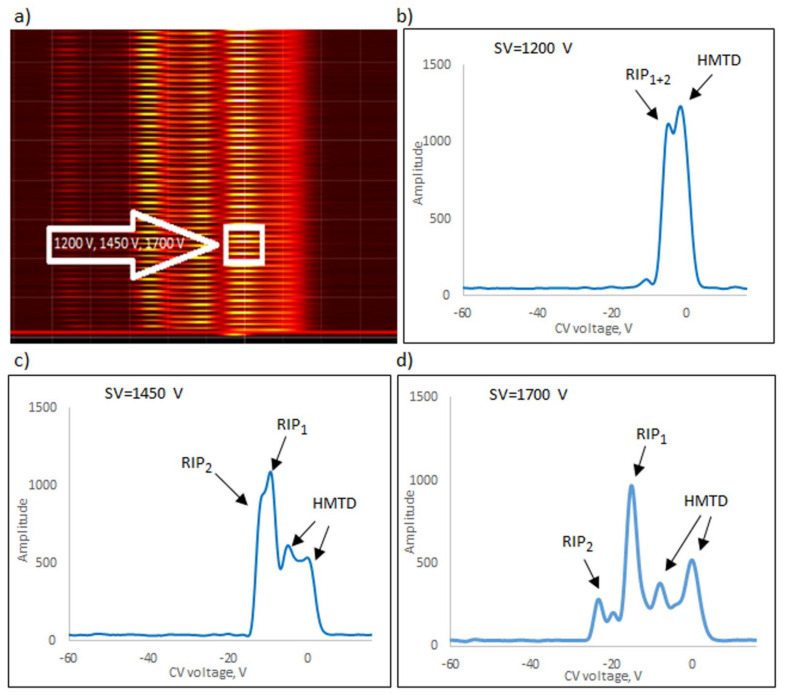
Dispersion plots for positive HMTD ions in (**a**) “multi” mode for voltages 1200 V, 1450 V, and 1700 V and in drift spectra for voltages (**b**) 1200 V, (**c**) 1450 V, and (**d**) 1700 V.

The following explosives/explosive taggants were tested using a stationary explosive trace detection system (a gate): NM, TATP, HMTD, DMDNB, and 4NT. Registration of dispersion plots was carried out in “multi” mode (cyclic data were read for strictly defined separation voltages (SVs)). Figure 9b–d, Figure 10b–d, Figure 11b–d, Figure 12b–d and Figure 13b–d respectively show the spectra extracted for specific SV values, which were characteristic of specific substances. In our case, these were three SVs: 1200 V, 1450 V, and 1700 V (Figure 9a, Figure 10a, Figure 11a, Figure 12a and Figure 13a respectively). One scan for these three SVs took approximately 1 s.

The following ions were visible on the spectra: reactant ion positive RIP_1_ (H^+^(H_2_O)_2–3_) and RIP_2_ (H^+^(NH_4_)). The positive reactant ions interacted with the analyte molecules (M), producing the protonated molecules MH^+^ (monomer ions). These monomer ions reacted with another molecule of the analyte to form proton-bound dimer ions, M_2_H^+^.

In the case of increased water vapor, the monomers move toward the higher negative values of the higher compensation voltages in the mode without a semi-permeable membrane. In the case of the mode with a semi-permeable membrane, it is necessary to apply a peak position correction due to the variable value of wetness.

The detection of explosives/explosive taggants consisted of scanning to identify the position of positive and negative ion peaks for three selected SVs (1200, 1450, and 1700 V). TATP, 4NT, NM, and DMDNB were identified using a detector with a semi-permeable membrane and a spectrometer without a membrane. HMTD was the exception; a detector without a membrane was used to detect HMTD ions.

The scaling structure was as follows:

- SV 1200 V, positive polarity—peak positions, CV voltage;
- SV 1200 V, negative polarization—peak positions, CV voltage;
- SV 1450 V, positive polarization—peak positions, CV voltage;
- SV 1450 V, negative polarity—peak positions, CV voltage;
- SV 1700 V, positive polarity—peak positions, CV voltage;
- SV 1700 V, negative polarity—peak positions, CV voltage.

Data were tested for each incoming data packet for both DMSs.

During testing, the most significant voltage was 1700 V, and the spectra were recorded for positive ions. The exception was NM, for which, for SV = 1700 V, the peak amplitude significantly decreased and the recorded peak shifted towards RIP; hence, it was necessary to use SV = 1450 V as the most relevant criterion. The voltages of 1200 V and 1700 V during the identification of this compound were only auxiliary.

When an explosive was detected, the gate entered cleaning mode. The time to restore the apparatus to operation was generally 10 min.

During analysis, peaks shifted depending on air humidity; this could cause errors in explosives identification. To guard against this, the analyzed air’s humidity was continuously measured, and peak positions were regularly corrected.

Table 1 presents the compensating voltage for positive ions at SV = 1700 V and 1450 V for NM.

## 4. Conclusions

Using a stationary system to detect trace amounts of explosives will be a powerful tool for ensuring state security and countering terrorist threats.

The system presented has many advantages, the most important of which are: an analysis time of up to 5 s. The gate is capable of detecting vapors and particulates (aeorosol) and identifying the type of explosive detected. The stationary system has a self-calibration and cleaning algorithm after the detection of explosive material. It is a maintenance-free system—the only necessary supervision is the flow of test personnel. It has a fairly low power consumption of (<300 W) in gas system temperature-maintenance mode, with a starting power of 3 kW (required to heat up the sample line). A very important element of the gate is also the ability to test without the need for a test-person confinement system or volume restriction for the test person.

The DMS detectors used in the gateway are characterized by high sensitivity, a low complexity of the gas system for the inlet path to the detectors, and a short purification time after detection. Fairly low selectivity necessitates the need to confirm explosive detection by swabbing the subject and testing on another device.

This stationary explosive detection system can easily be adapted to various types of traffic routes to test people at airports or border crossings.

## Figures and Tables

**Table 1:** Compensating voltage values for positive ions.

Substance	SV [V]	Positive Ion	CV [V]
4NT	1700	Monomer	−7.5
		Dimer	−3.1
NM	1450	Monomer	−10.7
		Dimer	-
DMDNB	1700	Monomer	−9.8
		Dimer	−0.4
TATP	1700	Monomer	−7.5
		Dimer	0.4
HMTD	1700	Monomer	−7.7
		Dimer	0.2

## Data Availability

Not applicable.
